# Serum ferritin as a non-invasive prognostic biomarker in cancer: a systematic review and meta-analysis

**DOI:** 10.3389/fonc.2026.1892172

**Published:** 2026-08-04

**Authors:** Xuewen Chen, Nan Bu, Qianying Lv, Junfang Zhao

**Affiliations:** Department of Clinical Laboratory, Tianjin Academy of Traditional Chinese Medicine Affiliated Hospital, Tianjin, China

**Keywords:** cancer, iron metabolism, non-invasive biomarker, overall survival, prognoses, serum ferritin

## Abstract

**Purpose:**

Serum ferritin (SF) is a routine biomarker reflecting iron status and systemic inflammation. We performed a systematic review and meta-analysis to validate the prognostic value of circulating SF for overall survival (OS) in adult patients with malignant tumors.

**Experimental design:**

We searched PubMed, Embase, Cochrane Library, the Chinese databases CNKI and Wanfang from inception to December 31, 2025, for retrospective cohort studies reporting the association between SF levels and overall survival (OS) in cancer patients. Two reviewers independently screened studies, extracted data, and assessed quality via the Newcastle-Ottawa Scale (NOS). Pooled hazard ratios (HRs) with 95% confidence intervals (CIs) were calculated, with prespecified subgroup, sensitivity, and publication bias analyses. This study was prospectively registered in PROSPERO (CRD420251271950).

**Results:**

Ten moderate-to-high quality studies (3,083 patients) were included. Elevated SF was significantly associated with increased mortality risk (pooled HR = 1.78, 95% CI: 1.55–2.05, *P* < 0.001), with consistent effects across all prespecified subgroups (all *P* for interaction > 0.05). We preliminarily identified a 300–500 ng/mL SF threshold as the high-risk range for adverse outcomes (exploratory analysis, 2 studies, HR = 2.26). Publication bias adjustment did not alter the core conclusion (adjusted HR = 1.70).

**Conclusion:**

Higher SF correlates with worse OS in adult patients with both hematologic malignancies and solid tumors. Our data only offers preliminary reference for risk stratification, and we cannot recommend routine clinical application right now. Larger prospective trials are needed to verify the ferritin concentration cut-off and different predictive values of pre- and post-treatment testing.

**Systematic Review Registration:**

https://www.crd.york.ac.uk/PROSPERO/, identifier CRD420251271950.

## 1 Introduction

Cancer remains a leading cause of mortality worldwide, accounting for approximately 10 million deaths annually and representing one in six total deaths globally (1). Among 177 countries, cancer ranks among the top three causes of premature mortality in adults aged 30 to 70 years (2), driving substantial reductions in life expectancy and imposing a growing societal and economic burden worldwide. With global cancer incidence projected to rise to 35 million new cases by 2050 (1), there is an urgent unmet clinical need for readily accessible, noninvasive biomarkers to refine prognostic risk stratification and enable personalized cancer care. Current prognostic strategies, which largely rely on invasive tissue biopsy and static clinicopathological features, are often suboptimal for dynamic disease monitoring and may not be feasible in patients ineligible for repeated tissue sampling.

As an easily measurable, low-cost, and noninvasive biomarker, SF is intimately associated with inflammatory responses, oxidative stress, and aberrant iron metabolism within the tumor microenvironment (3). It additionally plays a pivotal role in facilitating tumor angiogenesis, cellular proliferation, and immune regulation (4–7). These biological activities implicate SF as a potential integrative marker of the tumor-host interaction, reflecting both iron status and the systemic inflammatory milieu that shapes cancer progression. A growing body of evidence in recent years has substantiated that elevated SF levels are correlated with adverse prognosis across a diverse array of malignancies, including breast, colorectal, hepatocellular, lung, and prostate cancer, as well as B-cell lymphoma, peripheral T-cell lymphoma, and acute myeloid leukemia (8–16).

However, previous meta-analyses on this topic have unresolved critical limitations that limit its real-world clinical utility. Most focused exclusively on pre-treatment SF levels, ignoring the potential dynamic prognostic value of post-treatment measurements (17); few performed detailed stratified analyses of SF concentration gradients to identify clinically actionable risk thresholds; and none comprehensively addressed the heterogeneity in cut-off definitions that has driven inconsistent clinical conclusions. As a result, SF remains a research-only biomarker, with no standardized role in routine cancer prognostic assessment.

Hence, we performed this systematic review and meta-analysis, adhering to the Preferred Reporting Items for Systematic Reviews and Meta-Analyses (PRISMA) 2020 guidelines, with a prespecified protocol registered with the International Prospective Register of Systematic Reviews (PROSPERO), to comprehensively evaluate the prognostic value of SF across the entire cancer disease course. We designed this meta-analysis to answer four questions: (1) figure out whether ferritin levels relate to patient survival, noting that original studies used different adjustment factors so we cannot confirm fully independent effects; (2) test if this correlation stays stable in different subgroups; (3) explore potential ferritin risk cut-offs, but these subgroup results are only for reference due to limited sample size; (4) supply basic evidence for follow-up clinical research on ferritin biomarkers.

## 2 Materials and methods

### 2.1 Study design and data sources

This systematic review and meta-analysis were performed in strict adherence to the PRISMA 2020 reporting guidelines (18–20). The study protocol was prospectively registered in PROSPERO (registration number: CRD420251271950) prior to study initiation. We performed systematic literature searches in five electronic databases: PubMed (RRID: SCR_004846), Embase (RRID: SCR_001650), the Cochrane Library (RRID: SCR_013000), China National Knowledge Infrastructure (CNKI), and Wanfang data, from database inception to December 31, 2025. To identify potentially missed eligible studies, we supplemented the electronic searches with manual screening of the reference lists of included studies and relevant systematic reviews. The full, database-specific search strategies are provided in the **Supplementary Materials**.

All data used in this meta-analysis were extracted from previously published, peer-reviewed studies. All included original studies received ethical approval from their respective institutional review boards and obtained written informed consent from enrolled participants. Therefore, additional ethical approval and patient consent for this secondary analysis were not required.

Our literature search has several limitations. We conducted database searches across only five widely used Chinese and English electronic databases, without capturing grey literature, conference abstracts or unpublished trial data. Moreover, we only manually reviewed reference lists from the ten ultimately included studies, rather than every systematic review identified in our search. This practice may lead us to overlook relevant eligible studies and create selection bias within our analysis.

### 2.2 Inclusion and exclusion criteria

The eligibility criteria were prospectively predefined in accordance with the PECOS (Population, Exposure, Comparator, Outcome, Study design) framework, consistent with the registered study protocol.

Population: Patients aged ≥18 years with histologically or cytologically confirmed malignant tumors, with complete clinical and prognostic follow-up data available.

Exposure: Elevated circulating SF level (regardless of pre-treatment or post-treatment blood sampling timing), with the cut-off value predefined by each original study.

Comparator: Patients with lower SF levels from the same study cohort served as the control group.

Outcome: The primary endpoint was OS, defined as the time from cancer diagnosis or treatment initiation to all-cause death or the last follow-up. Eligible studies must report extractable HRs and corresponding 95% CIs for the prognostic association between SF and OS.

Study design: Retrospective cohort studies reporting the prognostic association between circulating SF levels and survival outcomes in patients with malignancies, and reporting extractable multivariate-adjusted hazard ratios.

Studies meeting any of the following criteria were excluded:

1. Secondary studies: systematic reviews, meta-analyses, narrative reviews, editorials, comments, and letters to the editor;
2. Non-clinical studies: preclinical animal experiments, *in vitro* cell studies, case reports, and case series with <10 patients;
3. Ineligible study design: cross-sectional studies, non-cohort clinical studies, and studies without a parallel control group;
4. Non-target exposure: studies measuring tissue ferritin instead of circulating serum ferritin;
5. Incomplete data: studies that did not report SF levels or lacked extractable HRs and 95% CIs for OS.

Since this meta-analysis solely covered non-interventional observational research, we used the PECOS framework instead of PICOS when developing our study inclusion criteria.

### 2.3 Study quality assessment and data extraction

Literature screening, data extraction, and methodological quality assessment were performed independently and in duplicate by two trained reviewers. Any disagreements were resolved through consensus discussion; Unresolved discrepancies were adjudicated by a third senior investigator with specialized expertise in cancer prognostic biomarker meta-analyses.

The risk of bias for each included retrospective cohort study was evaluated using the Newcastle–Ottawa Scale (NOS) (21), which evaluates study quality across three core domains: participant selection, cohort comparability, and outcome ascertainment. Studies with an NOS score ≥6 were defined as moderate to high quality.

Predefined data elements were systematically extracted from each eligible study: (1) basic study characteristics (first author, publication year, country, study period, sample size, follow-up duration); (2) baseline participant characteristics (age, gender, tumor type, tumor stage); (3) exposure details (SF sampling timing [pre-/post-treatment], cut-off value for elevated ferritin); (4) prognostic outcome data (HRs and 95% CIs for OS associated with elevated SF, adjusted for clinicopathological confounders).

For the 2 included Chinese-language studies, full-text articles were translated into English by two independent reviewers, with cross-validation of all extracted data and quality assessment scores to ensure accuracy.

### 2.4 Statistical analysis

All statistical analyses were performed using Review Manager version 5.4 (Cochrane Collaboration, Oxford, UK) and Stata 18.0. The primary endpoint was OS, and the prognostic association between circulating SF levels and OS was quantified by pooling HRs and corresponding 95% CIs from each eligible study. HRs and their standard errors were either directly extracted from the original publications’ multivariate Cox regression analyses or calculated from reported 95% CIs, two-sided P values, or Kaplan-Meier survival curves using the validated Tierney method (22). All HR estimates were log-transformed prior to meta-analysis to stabilize variance and approximate a normal distribution.

Between-study heterogeneity was assessed using the Cochrane Q test and quantified with the I² statistic (22). An I² value of 0% was defined as no heterogeneity, with values of 25%, 50%, and 75% corresponding to low, moderate, and high heterogeneity, respectively. A fixed-effects model was used for pooled analysis when *I²* < 50% and P > 0.10 for the Q test (no significant heterogeneity); a random-effects model was applied when significant between-study heterogeneity was detected (I² ≥ 50% or P ≤ 0.10) (17).

Leave-one-out sensitivity analysis was performed by sequentially omitting each individual study to verify the robustness and reliability of the pooled results. Prespecified subgroup analyses, consistent with the registered PROSPERO protocol, were conducted according to SF sampling timing (pre-treatment vs post-treatment), age (≥60 years vs <60 years), geographic region (Asian vs European populations), SF cut-off value (≥500 ng/mL vs <500 ng/mL), ferritin concentration gradient, and tumor type (hematologic malignancies vs solid tumors). Between-subgroup differences were evaluated via interaction tests, with a *P* value <0.05 defined as statistically significant.

Potential publication bias was assessed visually via funnel plots and quantitatively evaluated using Egger’s linear regression test. The trim-and-fill method was applied to adjust for potential publication bias if indicated by a significant Egger’s test result (*P* < 0.05).

### 2.5 Data availability

The data analyzed in this study were obtained from previously published peer-reviewed studies, all of which are cited in the reference list. All extracted individual study data and the complete Review Manager 5.4 project file (.rm5) are available from the corresponding author upon reasonable request. The full PRISMA 2020 checklist is provided in **Supplementary Materials**.

## 3 Results

### 3.1 Study selection

**Figure 1:**
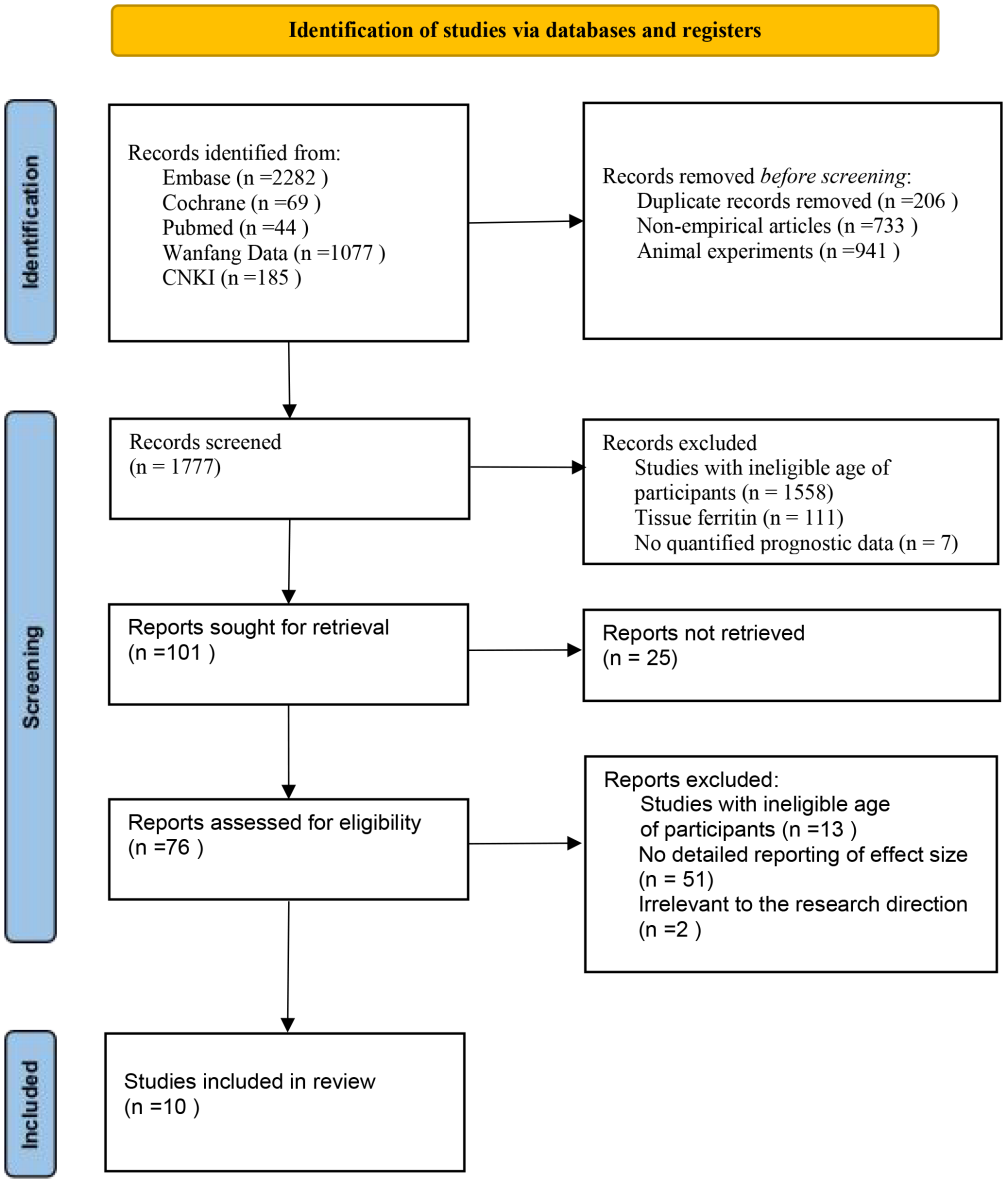
PRISMA 2020-compliant flow diagram of the literature search and study selection process for this systematic review and meta-analysis.

The study selection process was performed in strict accordance with the PRISMA 2020 statement, with the process and results detailed in the PRISMA flow diagram (**Figure 1**). The initial systematic literature search retrieved a total of 3657 records from five electronic databases. After removing 206 duplicate records, 733 non-empirical articles, and 941 animal studies, 1777 unique records remained for title and abstract screening. During this stage, 1676 ineligible records were excluded, and 25 additional records were excluded due to unavailable full text. The remaining 76 full-text articles underwent duplicate independent eligibility assessment. Finally, 66 studies were excluded for failing to meet the full predefined eligibility criteria, with 10 studies ultimately deemed eligible for inclusion in the final quantitative meta-analysis (23–32).

### 3.2 Characteristics and quality of included studies

All 10 included studies were retrospective cohort studies, conducted between 2009 and 2025 across six countries (South Korea, Germany, China, Japan, Spain, and Austria), enrolling a total of 3083 cancer patients (age range: 18–94 years; 2025 male, 1058 female patients). NOS scores ranged from 6 to 8, indicating all studies were of moderate to high methodological quality (**Table 1**).

**Table 1:** Methodological quality assessment of the 10 included retrospective cohort studies using the Newcastle-Ottawa Scale (NOS).

Study	Selection				Comparability	Outcome			Total score
	Representativeness of the exposed cohort	Selection of the non-exposed cohort	Ascertainment of exposure	Demonstration that outcome of interest was not present at start of study	Comparability of cohorts on the basis of the design or analysis	Assessment of outcome	Was follow-up long enough for outcomes to occur	Adequacy of follow up of cohorts	
Moo-KonSong.2009 (23)	1	1	1	1	1	0	1	1	7
Jana Ihlow,2019 (24)	1	1	1	1	1	1	1	1	8
Zetan Chen,2024 (25)	1	1	1	1	1	1	1	1	8
Shohei Kikuchi,2012 (26)	1	1	1	1	1	0	0	1	6
Gloria Moreno Carrasco,2025 (27)	1	1	1	1	1	1	1	1	8
Hiroshi Kawabata,2019 (28)	1	1	1	1	1	1	1	1	8
Kathrin Strasser-Weippl,2014 (29)	1	1	1	1	1	1	1	1	8
Daniel A. Quintana Pacheco,2018 (30)	1	1	1	1	2	1	1	0	8
Shiming Gu,2022 (31)	1	1	1	1	1	1	1	1	8
Jingyuan Zhao,2021 (32)	1	1	1	1	1	1	0	1	7

The NOS scale contains 3 domains (study selection, comparability, and outcome assessment) with a total maximum score of 9; studies with a total score ≥6 were defined as moderate to high quality. All included studies met this quality threshold.

For SF measurement, eight studies assessed pre-treatment circulating ferritin levels, and two studies measured post-treatment levels. Cut-off values for elevated SF varied across studies: two used the upper limit of normal (ULN) (23, 32), two used the interquartile range (IQR) (24, 30), One used the tertile median (25), one referenced clinical guidelines for iron overload diagnosis (26), one adopted a previously published threshold (27), one used the study-specific median (28), one used the study-specific mean (29), and one determined the optimal cut-off via receiver operating characteristic (ROC) curve analysis (31).

All studies (23–32) reported OS as the primary endpoint, with multivariate Cox regression analyses adjusting for key clinicopathological confounders. Full baseline characteristics of included studies are detailed in **Table 2**.

**Table 2:** Baseline characteristics of the 10 included retrospective cohort studies.

First author, year	Study type	Country	Timing of ferritin measurement	Ferritin cut-off determination	Ferritin cut-off value	Sample size	Gender (male/female)	Age(median or mean ± SD)	Reported outcomes
Moo-KonSong.2009 (23)	RC	Korea	Post-treatment	ULN	300 ng/mL	89	Male:46 Female:43	64(38-87)	OS
Jana Ihlow,2019 (24)	RC	Germany	Pre-treatment	IQR	750 ng/mL	137	Male:73 Female:64	54 ± 14.9	OS, RFS
Zetan Chen,2024 (25)	RC	China	Pre-treatment	Tertile median	164 ng/mL	252	Male:177 Female:75	49(19-80)	OS, PFS
Shohei Kikuchi,2012 (26)	RC	Japan	Pre-treatment	Clinical guideline for iron overload diagnosis	500 ng/mL	47	Male:28 Female:19	66(27-86)	OS
Gloria Moreno Carrasco,2025 (27)	RC	Spain	Pre-treatment	prior studies	1000 ng/mL	190	Male:133 Female:57	70(40-90)	OS, OR
Hiroshi Kawabata,2019 (28)	RC	Japan	Pre-treatment	the median serum ferritin level	210 ng/mL	107	Male:72 Female:35	70(20-94)	OS
Kathrin Strasser-Weippl,2014 (29)	RC	Austria	Pre-treatment	Average	250 ng/mL	137	Male:80 Female:57	56(27-72)	OS, PFS
Daniel A. Quintana Pacheco,2018 (30)	RC	Germany	Pre-treatment	IQR	quartiles	1632	Male:999 Female:633	63.0 ± 7.6	OS
Shiming Gu,2022 (31)	RC	China	Pre-treatment	ROC	108.63 ng/mL	112	Male:78 Female:34	50.13 ± 4.24	OS
Jingyuan Zhao,2021 (32)	RC	China	Post-treatment	ULN	Male: 200 ng/mL Female: 150 ng/mL	380	Male:339 Female:41	54(21-86)	OS

IQR, interquartile range; OR, Overall response rate; OS, Overall survival; PFS, Progression-free survival; RC, Retrospective cohort; ROC, receiver operating characteristic; RFS, Relapse-free survival; ULN, Upper limit of normal.

### 3.3 Primary prognostic association between serum ferritin and overall survival

**Figure 2:**
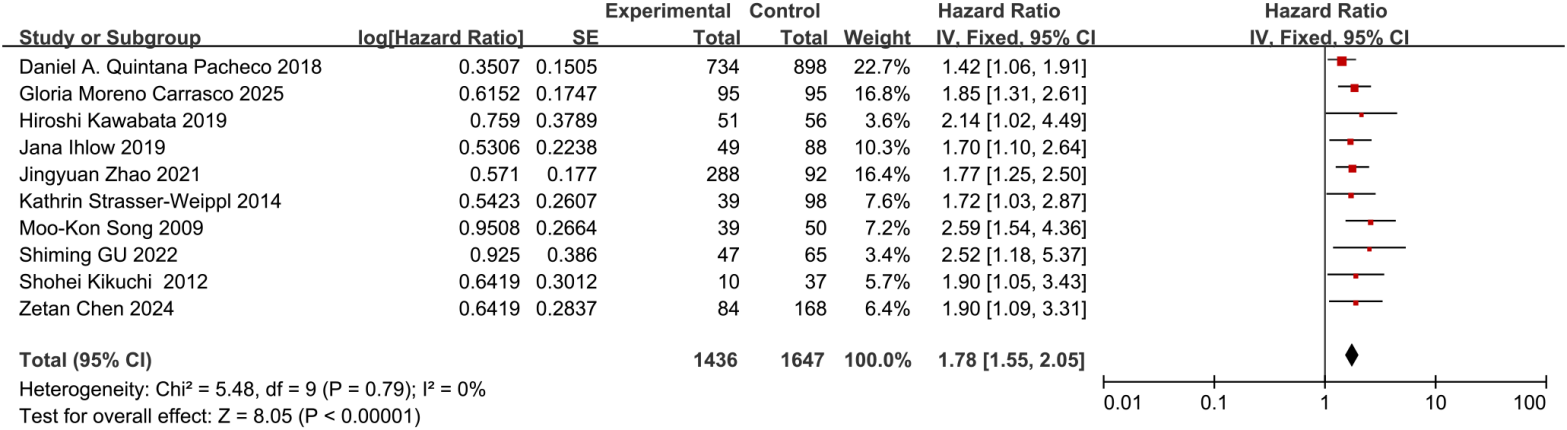
Forest plot of the pooled association between elevated circulating serum ferritin (SF) levels and overall survival (OS) in patients with cancer. Hazard ratios (HRs) and 95% confidence intervals (CIs) for individual studies and the overall pooled estimate are presented, with a fixed-effects model used for meta-analysis (between-study heterogeneity, *I²* = 0%).

Pooled fixed-effects analysis revealed that elevated circulating SF levels were significantly associated with an increased risk of all-cause mortality in adult cancer patients (pooled HR = 1.78, 95% CI: 1.55–2.05, *P* < 0.001; **Figure 2**). No significant between-study heterogeneity was detected in the primary analysis (*I²* = 0%, *P* for Cochrane Q test = 0.79).

### 3.4 Sensitivity analysis

Leave-one-out sensitivity analysis confirmed the robustness of the primary findings. Sequential omission of each individual study did not alter the direction, magnitude, or statistical significance of the pooled HR (range: 1.73–1.90; all *P* < 0.05), with between-study heterogeneity consistently remaining at *I²* = 0%. No single study was identified as the dominant driver of the overall pooled result.

### 3.5 Prespecified subgroup analyses

To explore potential sources of heterogeneity and validate the stability of the prognostic association between SF and OS, we performed a series of prespecified subgroup analyses, consistent with our registered PROSPERO protocol.

#### 3.5.1 By timing of SF measurement

Elevated SF was consistently associated with reduced OS in both pre-treatment (8 studies: HR = 1.72, 95% CI: 1.47–2.02, *P* < 0.001; *I²* = 0%) and post-treatment sampling subgroups (2 studies, exploratory analysis: HR = 1.99, 95% CI: 1.49–2.65, *P* < 0.001; *I²* = 29%). No significant between-stratum difference was detected (*P* for interaction = 0.39). Subgroup sensitivity analysis revealed mild heterogeneity between subgroups before and after treatment, but this did not lead to significant changes in the overall effect size.(Total *I²* = 0%).

Ferritin levels measured before and after treatment both predicted poorer survival outcomes. However, the post-treatment subgroup only included two small study cohorts. While hazard ratios appeared higher for post-treatment ferritin, this observation does not confirm stronger prognostic value, so these findings require careful interpretation.

#### 3.5.2 By patient age, geographic region, and cut-off threshold

The prognostic association remained statistically significant across all strata, with no significant between-subgroup heterogeneity detected:

Age ≥60 years (5 studies: HR = 1.76, 95% CI: 1.46–2.12, *P* < 0.001) vs <60 years (5 studies: HR = 1.81, 95% CI: 1.47–2.24, *P* < 0.001; *P* for interaction = 0.83).

Asian populations (6 studies: HR = 2.01, 95% CI: 1.62–2.50, *P* < 0.001) vs European populations (4 studies: HR = 1.63, 95% CI: 1.35–1.96, *P* < 0.001; *P* for interaction = 0.14).

After excluding 1 study (30) without reported quantitative SF values, Cut-off ≥500 ng/mL (4 studies: HR = 1.93, 95% CI: 1.55–2.41, *P* < 0.001) vs <500 ng/mL (5 studies: HR = 1.87, 95% CI: 1.49–2.36, *P* < 0.001; *P* for interaction = 0.85).

#### 3.5.3 By SF concentration gradient (exploratory analysis)

After excluding 1 study (30) without reported quantitative SF values, elevated SF was consistently associated with increased mortality across all concentration strata, with no significant linear dose-response relationship detected (P for interaction = 0.62):

≤250 ng/mL (5 studies: HR = 1.87, 95% CI: 1.49–2.36, *P* < 0.001);

300–500 ng/mL (2 studies: HR = 2.26, 95% CI: 1.53–3.34, *P* < 0.001);

≥750 ng/mL (2 studies: HR = 1.79, 95% CI: 1.37–2.35, *P* < 0.001).

We found the 300–500 ng/mL subgroup yielded the largest pooled hazard ratio. Still, this exploratory subgroup analysis relied on merely two independent studies, and we detected no significant interaction via interaction testing. For this reason, this ferritin threshold cannot yet serve as an official clinical risk cutoff.

#### 3.5.4 By cancer type

Elevated SF was significantly associated with increased mortality risk in both hematologic malignancies (6 studies: HR = 1.91, 95% CI: 1.57–2.33, *P* < 0.001; *I²* = 0%) and solid tumors (4 studies: HR = 1.65, 95% CI: 1.35–2.02, *P* < 0.001; *I²* = 0%). No significant between-stratum difference was observed (P for interaction = 0.31).

**Figure 3:**
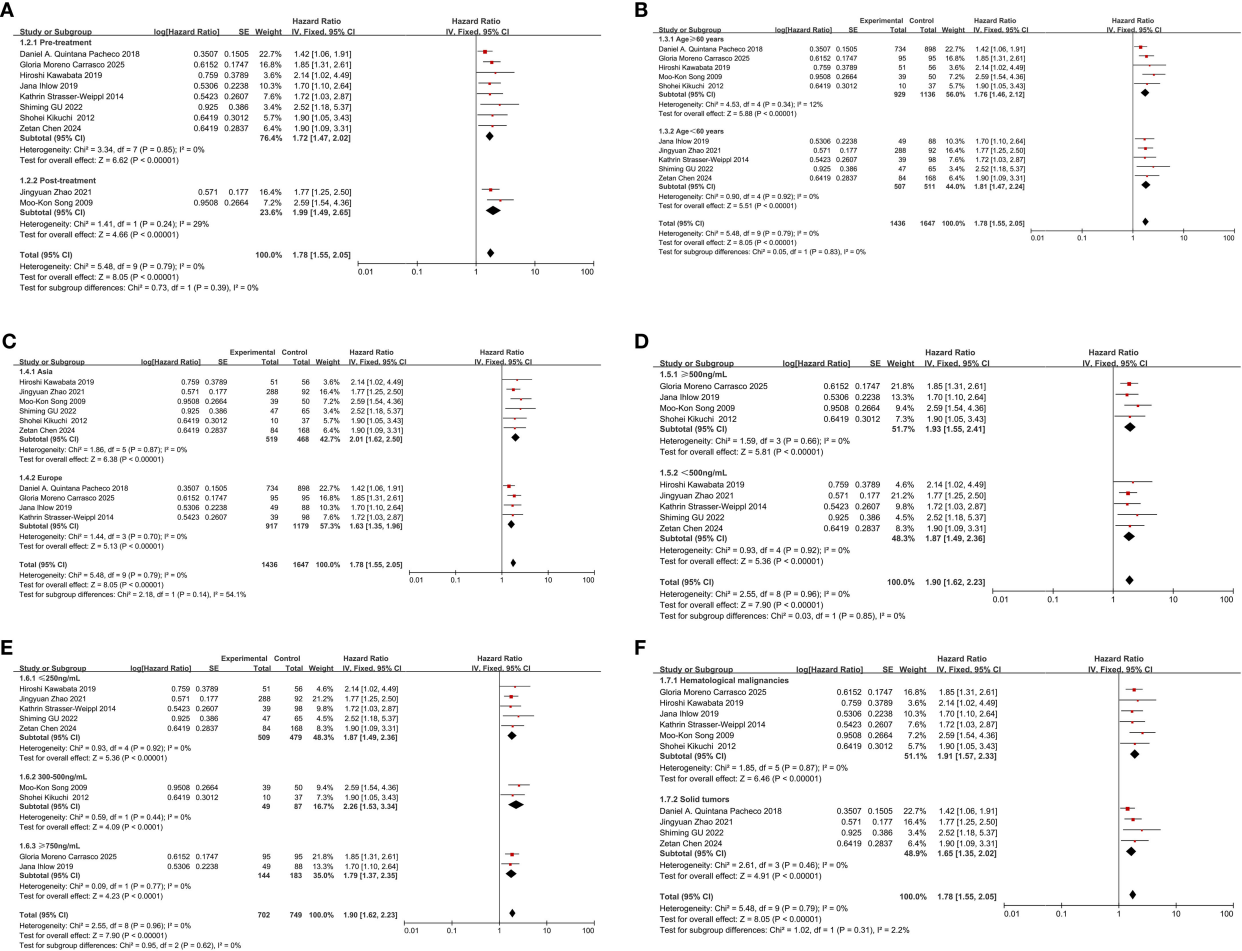
Forest plot of the association between SF levels and OS in patients with cancer across different subgroups. **(A)** Forest plot of prespecified subgroup analysis comparing the prognostic association between pre-treatment and post-treatment SF levels and OS in patients with cancer. Pooled HRs, 95% CIs, and *I²* are reported for each subgroup; post-treatment analysis is exploratory (2 studies). Post-treatment subgroup only includes two small cohorts; the seemingly stronger correlation is preliminary and cannot be taken as definitive evidence. **(B)** Forest plot of prespecified subgroup meta-analysis of the association between elevated SF levels and OS in patients with cancer, stratified by age (≥60 years vs <60 years). Pooled HRs, 95% CIs, and *I²* values are presented for each stratum. **(C)** Forest plot of prespecified subgroup meta-analysis of the association between elevated SF levels and OS in patients with cancer, stratified by geographic region (Asian vs European populations). Pooled HRs, 95% CIs, and I² values are presented for each stratum. **(D)** Forest plot of prespecified subgroup meta-analysis of the association between elevated SF levels and OS in patients with cancer, stratified by SF cut-off threshold (≥500 ng/mL vs <500 ng/mL). Pooled HRs, 95% CIs, and I² values are presented for each stratum. **(E)** Forest plot of prespecified subgroup meta-analysis of the association between SF concentration gradients and OS in patients with cancer, stratified by pre-specified concentration intervals (≤250 ng/mL, 300–500 ng/mL, and ≥750 ng/mL). Pooled HRs, 95% CIs, and I² values are presented for each interval. This 300–500 ng/mL subgroup analysis is exploratory with just two studies; this cut-off is not suitable for clinical risk judgment. **(F)** Forest plot of prespecified subgroup meta-analysis of the association between elevated SF levels and OS in patients with cancer, stratified by cancer type (hematological malignancies vs solid tumors). Pooled HRs, 95% CIs, and I² values are presented for each stratum.

Full forest plots for all subgroup analyses are provided in **Figures 3A-F**.

#### 3.5.5 By male proportion of included cohorts

We further performed a *post-hoc* subgroup analysis stratified by the median proportion of male patients in each cohort (cut-off: 67% males, **Supplementary Figure 1**). One study lacked gender composition data and was excluded from this stratified analysis, leaving nine studies eligible for comparison.

We carefully checked all ten included articles. No paper reported separate sex-specific hazard ratios for serum ferritin. Only two studies adjusted gender in their multivariate Cox models, yet neither published male-only or female-only effect estimates. Without individual patient data, we could not perform a meta-analysis stratified by each patient’s sex. Instead, we conducted a *post-hoc* subgroup analysis based on the overall proportion of men within each study cohort.

After excluding 1 study (29) without reported gender distribution, nine studies remained for this stratified subgroup analysis.

≥67% male patients (5 studies: HR = 1.89, 95% CI: 1.54–2.32, *P* < 0.001; *I²* = 0%);

<67% male patients (4 studies: HR = 1.68, 95% CI: 1.37–2.07, *P* < 0.001; *I²* = 26%);

The test for subgroup difference showed no statistically significant difference between the two groups (*P* = 0.44).

This subgroup analysis was not predefined in our original protocol and should be treated as exploratory only. We did not set the 67% male cut-off before data extraction; this threshold was chosen based on the median proportion of male patients across all evaluable studies.

#### 3.5.6 By adjustment for sex

We conducted an extra *post-hoc* subgroup analysis to assess whether controlling for sex as a covariate would shift the pooled hazard ratio(**Supplementary Figure 2**).

Among the two studies that adjusted for sex, the combined HR was 1.99 (95% CI: 1.25–3.16, *P* = 0.004; *I²* = 0%). The seven studies without sex adjustment yielded a comparable pooled HR of 1.76 (95% CI: 1.51–2.06, *P* < 0.001; *I²* = 0%).

We observed no statistically significant disparity between these two subgroups (*χ²* = 0.23, df = 1, *P* = 0.63). This finding confirms that sex adjustment did not materially change our main effect estimate.

### 3.6 Supplementary meta-regression analysis

#### 3.6.1 Single-variable meta-regression was performed to assess predefined covariates

Single-variable meta-regression was performed to evaluate six predefined moderators of heterogeneity, with overall survival HRs as effect sizes analyzed on a natural log scale under a REML random-effects model. Examined covariates comprised cohort sample size, male proportion, publication year, serum ferritin cutoff value, blood sampling timing, and tumor type. Ratio of hazard ratios (RHR) and 95% CIs for each moderator are visualized in **Supplementary Figure 3**.

No covariate produced statistically significant regression coefficients, as all RHR 95% CIs crossed 1.0 and all *P* values > 0.05 (cohort size: RHR = 0.979, *P* = 0.073; male proportion: RHR = 0.995, *P* = 0.942; publication year: RHR = 0.967, *P* = 0.673; SF cutoff: RHR = 0.981, *P* = 0.477; sampling timing: RHR = 0.865, *P* = 0.390; tumor type: RHR = 1.155, *P* = 0.315). Only cohort size showed a marginal non-significant trend. In the current analysis, the modifying effects of the above factors on the association between elevated ferritin levels and poor OS were not statistically significant (**Supplementary Table 1**).

#### 3.6.2 Clinical subgroup analyses

We conducted clinical subgroup analyses alongside interaction tests to explore potential effect modification of the association between SF and OS (**Supplementary Table 2**).

When grouping studies by sampling timepoint, elevated ferritin remained a significant adverse prognostic marker in both baseline pre-treatment cohorts (8 studies, HR = 1.721, 95% CI 1.466–2.021, *P* < 0.001) and cohorts with ferritin measured during or after anti-tumor therapy (2 studies, HR = 1.989, 95% CI 1.491–2.655, *P* < 0.001); the interaction P was 0.390 for this stratification factor.

We further stratified included studies by tumor subtype. The pooled HR reached 1.911 (95% CI 1.570–2.325, *P* < 0.001) across six studies focusing on hematologic malignancies, while the pooled estimate stood at 1.654 (95% CI 1.353–2.023, *P* < 0.001) for four solid tumor investigations, with an interaction *P* of 0.315.

Elevated ferritin predicted worse OS uniformly across all clinical subgroups. All interaction *P* values were >0.05, demonstrating that sampling timing and tumor histology did not substantially modify the adverse prognostic link between ferritin and overall survival.

#### 3.6.3 Robustness analyses of moderator coding

We performed sensitivity analyses to verify whether alternative coding strategies for two continuous moderators altered the meta-regression inference (**Supplementary Table 3**).

For the moderator of male proportion, complete-case analysis included 9 studies, with a regression coefficient *β* =−0.002 (*P* = 0.969). The alternative median imputation model with missing indicators incorporated 10 studies and returned identical *β* and *P* values (*β* =−0.002, *P* = 0.969), supporting consistent effect direction and statistical inference across analytical frameworks.

For the SF cutoff moderator, the strict coding model (8 studies, *β* =−0.019, *P* = 0.477) and the alternative sensitivity model (9 studies, *β* =−0.011, *P* = 0.660) both failed to detect significant effect modification, consistent with the table conclusion that no meaningful moderating effect was observed under either coding scheme.

Across both moderators, varying the analytical approach for missing data or SF threshold definition did not change our core meta-regression conclusions.

### 3.7 Publication bias

**Figure 4:**
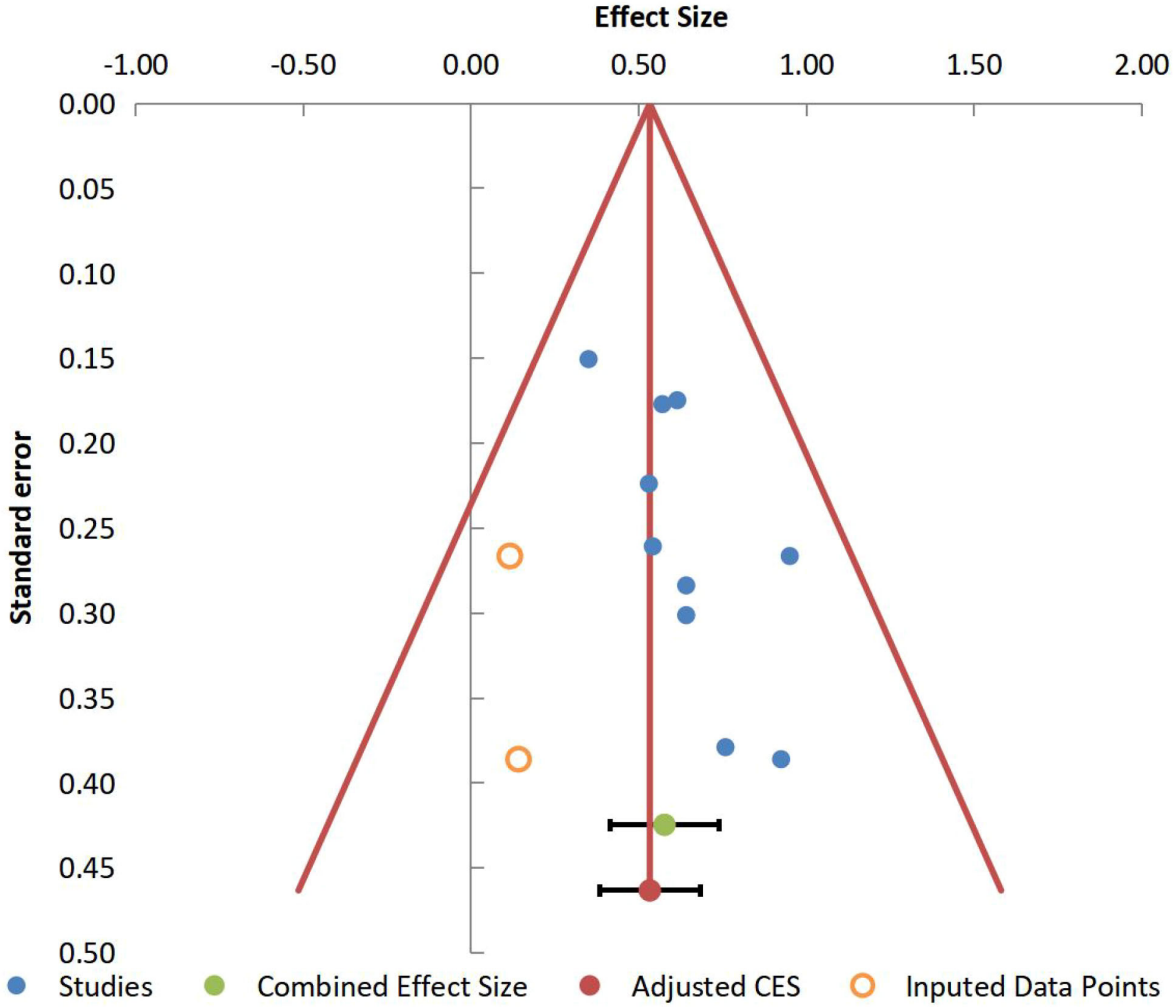
Funnel plot of the pooled HRs for OS to assess potential publication bias in the meta-analysis. Asymmetry was evaluated via Egger’s linear regression test, with trim-and-fill adjustment performed to account for potential unpublished studies. Funnel plot shows publication bias via Egger’s test. After trim-and-fill correction, pooled HR decreased slightly, suggesting missing unpublished negative trials may affect our results.

Egger’s linear regression test indicated significant funnel plot asymmetry (*t* = 2.88, *P* = 0.020), suggesting potential publication bias. The trim-and-fill method was applied, estimating the imputation of 2 theoretically missing studies with negative outcomes. Following adjustment, the pooled HR decreased from 1.78 to 1.70 (95% CI: 1.48–1.95, *P* < 0.001), with the direction and statistical significance of the association unchanged. The funnel plot is provided in **Figure 4**.

## 4 Discussion

Our pooled results show elevated ferritin correlates with poorer OS across different cancers, a finding consistent with prior individual clinical studies (33–36). We adopted multiple statistical approaches to test the reliability of this primary correlation. Leave-one-out sensitivity analysis confirmed no single study drove the overall effect, between-study heterogeneity was negligible (*I²* = 0%), and adjustment for publication bias did not alter the core conclusion. We also conducted several supplementary *post-hoc* analyses, meta-regression and sensitivity tests for variable coding to evaluate potential confounding sources that might alter the SF–OS association. Even with these validations, we cannot confirm SF as an independent prognostic marker for cancer survival, given inconsistent covariate adjustment across included retrospective studies and a lack of individual patient data (IPD).

Varied SF cutoff definitions are widely recognized as a major source of inconsistent prognostic results in previous literature (35). The cohorts we included adopted different thresholds to define high ferritin, including upper limits of normal, interquartile ranges, median values and ROC-derived optimal cutoffs. Regardless of which threshold was applied, high SF remained significantly linked to worse survival. Notably, significant prognostic risk persisted even among patients with mildly elevated ferritin within standard reference intervals (≤250 ng/mL, HR = 1.87). A stronger hazard trend was observed in studies using a 300–500 ng/mL cutoff (HR = 2.26). These observations imply mild iron metabolism disturbances, not only pathological iron overload, carry prognostic meaning for cancer patients. Clinicians should therefore avoid relying solely on standard upper reference limits when interpreting SF levels. Nevertheless, this 300–500 ng/mL subgroup analysis relies on merely two small studies; we regard this finding as exploratory and do not recommend using this range for clinical risk stratification at present.

We further stratified studies by blood sampling timing and tumor type to explore potential effect modification. For sampling timepoints, elevated ferritin predicted unfavorable OS both in pretreatment cohorts (8 studies, HR = 1.721) and cohorts with measurements during or after anti-tumor therapy (2 studies, HR = 1.989), with a non-significant interaction *P* value of 0.390. The numerically higher HR in post-treatment cohorts may reflect cumulative tumor progression and therapy-induced systemic inflammation, as reported in earlier clinical work (37–39). Still, post-treatment ferritin is easily disturbed by chemotherapy-related inflammatory responses, transfusions and liver dysfunction, and the limited number of relevant studies restricts robust conclusions on its clinical utility for monitoring treatment response.

When grouping by tumor subtype, elevated SF predicted poorer survival in both hematological malignancies (6 studies, HR = 1.911) and solid tumors (4 studies, HR = 1.654), and the interaction test showed no meaningful subgroup difference (*P* = 0.315). This consistent correlational trend suggests ferritin-related inflammatory and iron metabolic changes may affect prognosis across diverse tumor histologies, though we lack direct clinical evidence to confirm a shared causal mechanism.

Gender represents a critical potential confounder in ferritin research. Physiologically, women tend to have lower baseline ferritin due to menstruation, pregnancy and lactation, while men generally maintain higher iron reserves. Most original cohorts did not report sex-stratified survival data, and only two studies incorporated gender into multivariate Cox models. To assess whether gender imbalance biased our pooled estimates, we designed two complementary *post-hoc* subgroup analyses focused on gender-related factors.

First, we stratified cohorts by the median male proportion of 67%. Significant adverse OS associations existed in both cohorts with ≥67% male patients (HR = 1.89) and cohorts with fewer male participants (HR = 1.68), and the subgroup difference test yielded *P* = 0.44. Second, we compared studies with versus without gender adjustment in multivariate models. The pooled HR was 1.99 for sex-adjusted studies and 1.76 for unadjusted studies, with no statistically significant between-group difference (*P* = 0.63). Taken together, these aggregate-level analyses tentatively suggest the overall SF–OS association is not driven primarily by male-heavy cohorts or unadjusted gender imbalance. It is important to emphasize that both analyses were unplanned *post-hoc* tests; the 67% male proportion cutoff was determined after data extraction. Moreover, stratification based on cohort-wide male percentages cannot capture individual-level sex differences in ferritin and survival, so residual gender confounding cannot be fully excluded without IPD.

We performed single-variable meta-regression to evaluate six predefined moderators that might explain between-study heterogeneity: cohort size, male proportion, publication year, SF cutoff value, sampling timing and tumor type. None of these covariates showed significant modifying effects (all *P* > 0.05), with only cohort sample size displaying a weak non-significant trend. We additionally carried out sensitivity analyses for the coding of two continuous moderators (male proportion and SF cutoff) to test whether different handling of missing data or threshold definitions altered regression results. Both complete-case and median imputation models for male proportion, as well as two alternative coding schemes for SF cutoffs, produced consistent non-significant regression coefficients. These robustness checks add supporting evidence that the null modifying effects of the above variables were not artifacts of our analytical setup.

Furthermore, the correlation between high ferritin and poor survival remained stable across different age groups and geographic regions, and no obvious subgroup differences were observed. These results only provide preliminary evidence that ferritin may have consistent predictive trends in various populations, but we cannot draw solid conclusions for widespread global clinical use given the multiple confounding factors we identified above.

Despite comprehensive supplementary statistical testing, our analysis has several notable limitations. All included studies were retrospective, which inherently introduces selection and recall bias. Egger’s test indicated potential publication bias, meaning small cohorts reporting neutral or non-significant SF survival associations may remain unpublished and shift our pooled hazard estimates. Most primary studies failed to adjust for key inflammatory markers such as CRP and IL-6. Since ferritin acts as an acute-phase reactant, the observed correlation with poor survival may partially reflect tumor-triggered systemic inflammation rather than a tumor-specific independent risk factor. Multiple exploratory subgroups (post-treatment sampling, 300–500 ng/mL ferritin) contained very few studies, making these trends unstable for clinical inference. One large cohort accounted for over half of all enrolled participants; although leave-one-out analysis confirmed this study did not dominate the overall result, uneven sample distribution requires cautious interpretation. Finally, our findings only apply to adult cancer patients and cannot be extended to children or patients with rare malignant diseases.

### 4.1 Underlying biological mechanisms

All mechanisms described below are derived solely from cell and animal experiments and provide theoretical context for our correlational meta-analysis results. No clinical data from this study can validate causal biological links between ferritin and tumor progression, so further translational laboratory research is required.

First, excess systemic iron may trigger oxidative DNA damage. High circulating ferritin signals iron overload, where free iron catalyzes reactive oxygen species (ROS) production via the Fenton reaction. Excess ROS may induce DNA double-strand breaks in tumor cells (40), accelerate oncogenic mutations, disrupt antioxidant defenses, and reduce tumor sensitivity to chemo- and radiotherapy. This iron-mediated oxidative pathway may partially explain the stronger prognostic trend seen in the 300–500 ng/mL subgroup, yet the small sample size of this subgroup prevents definitive causal inference.

Second, ferritin reshapes the tumor immune microenvironment to favor immunosuppression. Iron released from ferritin supports tumor cell proliferation, while also promoting polarization of tumor-associated macrophages toward an anti-inflammatory M2 phenotype. This process suppresses cytotoxic activity of CD8+ T cells and NK cells, ultimately boosting tumor invasion and metastatic potential (41). This immune-related pathway may partially account for consistent SF prognostic effects across hematological and solid tumors in our subgroup comparisons.

Third, ferritin participates in chronic tumor-promoting inflammation and epithelial-mesenchymal transition (EMT) (3). As an established acute-phase reactant (42), elevated ferritin correlates closely with elevated pro-inflammatory cytokines including IL-6. The sustained inflammatory microenvironment can activate EMT programs, enhancing tumor cell migration and invasion and worsening long-term survival. This pathway also offers a potential explanation for the numerically higher HR observed in post-treatment cohorts: anti-tumor therapy induces tumor cell death and iron release, aggravating systemic inflammation and secondary ferritin elevation. Current clinical evidence remains insufficient to confirm this sequential chain of events, and targeted preclinical and prospective cohort studies are needed to verify these molecular processes.

### 4.2 Clinical translational implications

Our findings deliver preliminary exploratory evidence supporting the prognostic relevance of serum ferritin across multiple malignancies, though the current dataset does not warrant its immediate routine clinical implementation as a standardized prognostic biomarker. As an affordable, widely available laboratory test routinely ordered in clinical practice (43), SF holds substantial potential as a prognostic indicator for cancer risk stratification (44). Multiple barriers, however, restrict its immediate clinical adoption: inconsistent testing protocols and cut-off values across studies preclude unified diagnostic standards; most original cohorts failed to fully adjust for sex and inflammatory confounders including CRP and IL-6, limiting confirmation of SF’s independent prognostic effect; additionally, the prominent risk signal within the 300–500 ng/mL range and elevated HRs for post-treatment SF rely on small exploratory subgroups without robust validation.

Collectively, existing data only identify a correlational link between high SF and poor OS, without evidence that SF predicts treatment response. We thus do not recommend integrating ferritin into formal staging systems or adjusting clinical management purely based on ferritin levels. Although aberrant iron metabolism is a viable preclinical therapeutic target, our meta-analysis cannot provide sufficient clinical evidence to support iron-modulating agents for cancer treatment.

Even so, SF retains meaningful translational potential with targeted follow-up research. Future large prospective cohorts should establish uniform testing standards and population-specific cutoffs to validate the 300–500 ng/mL risk threshold and post-treatment SF monitoring. Individual patient-level analyses with comprehensive adjustment for sex and inflammatory biomarkers will further eliminate confounding bias. Upon resolving these limitations, SF could be deployed for routine cancer risk stratification, facilitating personalized follow-up and advancing translational research on iron-targeted anti-tumor therapies.

### 4.3 Strengths, novel contributions, and limitations

#### 4.3.1 Study strengths and novel contributions

This meta-analysis has several methodological strengths. We searched both Chinese and English databases with clear pre-defined inclusion and exclusion criteria. Beyond standard leave-one-out sensitivity and publication bias assessment, we performed multiple supplementary analyses: clinical subgroup stratification by sampling timing and tumor type, two *post-hoc* subgroup analyses addressing gender imbalance, single-variable meta-regression, and sensitivity tests for moderator coding. These additional analyses allowed us to systematically explore sources of between-study heterogeneity and test the consistency of our main result. Overall heterogeneity across all included cohorts was low (*I²* = 0%), indicating broadly consistent effect estimates across individual studies.

Compared with previous meta-analyses on ferritin and cancer survival, this work adds several exploratory observations requiring further validation. First, we found significant adverse survival associations regardless of ferritin cutoff definition, which may help reconcile conflicting results reported in earlier reviews. Second, separate stratification by treatment phase demonstrated prognostic value for both baseline and post-treatment ferritin, with a modestly higher hazard estimate in post-treatment cohorts. Third, consistent prognostic effects were observed in both hematological and solid tumor subgroups. Fourth, cohort-level subgroup analyses focused on male proportion and gender adjustment provided tentative evidence that gender imbalance does not fully explain the overall SF–OS correlation. Finally, we verified that different analytical approaches for missing data and SF threshold coding did not alter meta-regression outcomes.

#### 4.3.2 Study limitations

Multiple key limitations constrain the interpretability of our results. All ten included studies were retrospective cohorts, which carry inherent risks of selection and recall bias. Although we restricted inclusion to studies with moderate-to-high NOS scores and multivariate-adjusted HRs, large prospective multicenter cohorts are necessary to clarify potential causal relationships. Our literature search did not fully screen grey literature or reference lists of all relevant systematic reviews, which may omit unpublished neutral or negative studies and introduce selection bias. Egger’s test confirmed measurable publication bias, which may inflate the pooled hazard ratio.

Testing protocols and ferritin thresholds varied widely across studies, and uniform laboratory measurement standards are absent from current literature. Several exploratory subgroups contained only two studies each, leading to unstable effect estimates that we refrain from overinterpreting. One large cohort (30) contributed over half of all participants; while leave-one-out analysis excluded its dominant influence, this uneven sample distribution should be noted during result interpretation.

The most important limitation relates to gender confounding. No included study reported sex-stratified survival outcomes, and only two adjusted for gender in multivariate models. Although we conducted *post-hoc* subgroup analyses based on cohort male proportion and gender adjustment status, these aggregate-level comparisons cannot resolve baseline ferritin differences between individual men and women. Without IPD, we could not perform patient-level sex stratification to fully eliminate gender-related bias. Most original studies also failed to adjust for inflammatory markers such as CRP and IL-6, meaning the observed ferritin-survival association may reflect non-tumor-specific systemic inflammation. Lastly, our results cannot be generalized to children or patients with rare malignancies.

### 4.4 Future directions

Future research should address the unresolved limitations outlined above. Large prospective cohorts with standardized ferritin testing protocols are needed to establish unified, tumor-specific and treatment-phase-specific optimal cutoffs. Additional work should validate whether the 300–500 ng/mL ferritin range carries meaningful clinical risk stratification value, and clarify whether post-treatment ferritin monitoring offers advantages over baseline measurements. Basic mechanistic experiments are required to disentangle the separate contributions of iron metabolism dysregulation and inflammatory signaling to tumor progression.

Individual patient data meta-analysis with stratified sex-specific survival analyses should be prioritized to thoroughly account for physiological gender differences in baseline ferritin levels and survival endpoints. Future clinical cohorts should uniformly record gender, inflammatory biomarkers including CRP and IL-6, and other key confounders in multivariate prognostic models to better quantify ferritin’s independent prognostic effect. More studies covering rare tumors and pediatric cancer populations are also required to evaluate whether ferritin acts as a pan-cancer prognostic biomarker across broader patient populations.

## 5 Conclusions

This meta-analysis demonstrates that elevated serum ferritin is significantly associated with inferior overall survival in adult cancer patients. The prognostic association remained stable across different tumor types and diverse ferritin cutoff definitions, and was not substantially modified by sampling timing, tumor histology, cohort male proportion, or sex adjustment status. Meta-regression and moderator robustness analyses further confirmed that methodological and clinical variables exerted no significant modifying effects on the pooled association, supporting the overall statistical consistency of our primary findings.

Nevertheless, several critical limitations restrict the clinical translation of the present results. All included studies were retrospective with inconsistent covariate adjustment, and residual confounding from gender and systemic inflammation could not be fully resolved due to the absence of individual patient data. Exploratory subgroup analyses regarding post-treatment ferritin changes and the 300–500 ng/mL ferritin threshold were based on limited study numbers and remain unvalidated. In addition, evident publication bias may have influenced the pooled effect estimation.

In summary, current evidence only supports ferritin as an exploratory prognostic biomarker for cancer research rather than a reliable indicator for routine clinical risk evaluation. Future large prospective studies with standardized ferritin measurement protocols, unified cut-off values, individual-level sex stratification, and rigorous adjustment for inflammatory markers are warranted to validate our findings. Further basic mechanistic research is also needed to clarify ferritin’s distinct roles in iron metabolism and tumor inflammation, which may facilitate the development of iron-targeted anti-tumor strategies.

## Data Availability

The original contributions presented in the study are included in the article/**Supplementary Material**. Further inquiries can be directed to the corresponding authors.

## Supplementary material

Supplementary Figure 1 Forest plot of *post-hoc* subgroup analysis stratified by median proportion of male patients (cut-off 67%). This subgroup analysis was not prespecified in our review protocol and is exploratory. No significant difference was detected between the two subgroups (*P* = 0.44). One study without gender ratio data was excluded from this stratified analysis.

Supplementary Figure 2 Forest plot of *post-hoc* subgroup analysis stratified by adjustment for sex. This subgroup analysis was not prespecified in our review protocol and is therefore exploratory in nature. No statistically significant difference was observed between the two subgroups (*P* = 0.63).

Supplementary Figure 3 Forest plot of univariable meta-regression evaluating potential moderators of the association between elevated serum ferritin and overall survival. RHR, ratio of hazard ratio; CI, confidence interval. The vertical dashed line at RHR = 1 represents the null effect. All covariates exhibited non-significant modifying effects (all *P* > 0.05). The dashed line signifies the null value (RHR = 1). All 95% confidence intervals (CIs) encompassed the null value, indicating no statistically detectable effect modification by the prespecified moderators within the available study set.

Supplementary Table 1 Results of the univariate REML meta-regression.

Supplementary Table 2 Clinical subgroup analyses and interaction tests.

Supplementary Table 3 Robustness analyses of moderator coding.
