# Supplementary material for: Serum ferritin as a non-invasive prognostic biomarker in cancer: a systematic review and meta-analysis
Source: Front Oncol. 2026 Aug 4;16:1892172. doi: 10.3389/fonc.2026.1892172 (PMC13481198; doi:10.3389/fonc.2026.1892172)
Supplement: Supplementary file 1 [file SupplementaryFile1.pdf]

Supplementary Materials

Serum Ferritin as a Non-invasive Prognostic Biomarker in cancer: A Systematic Review and Meta-Analysis

Table of Contents

1. PRISMA 2020 Checklist

2. Full Database-Specific Search Strategies

3. Extracted Study Data

1. PRISMA 2020 Checklist

Section and Topic	Item #	Checklist item	Location where item is reported
**TITLE**			
Title	1	Identify the report as a systematic review.	Title
ABSTRACT			
Abstract	2	See the PRISMA 2020 for Abstracts checklist.	Abstract.
INTRODUCTION			
Rationale	3	Describe the rationale for the review in the context of existing knowledge.	Introduction.
Objectives	4	Provide an explicit statement of the objective(s) or question(s) the review addresses.	Abstract, Purpose.
METHODS			
Eligibility criteria	5	Specify the inclusion and exclusion criteria for the review and how studies were grouped for the syntheses.	Materials and Methods, Inclusion and Exclusion Criteria, Statistical Analysis.
Information sources	6	Specify all databases, registers, websites, organisations, reference lists and other sources searched or consulted to identify studies. Specify the date when each source was last searched or consulted.	Materials and Methods, Study Design and Data Sources.
Search strategy	7	Present the full search strategies for all databases, registers and websites, including any filters and limits used.	Materials and Methods, Study Design and Data Sources.
Selection process	8	Specify the methods used to decide whether a study met the inclusion criteria of the review, including how many reviewers screened each record and each report retrieved, whether they worked independently, and if applicable, details of automation tools used in the process.	Materials and Methods, Study Quality Assessment and Data Extraction, Statistical Analysis.
Data collection process	9	Specify the methods used to collect data from reports,	Materials and Methods,

Section and Topic	Item #	Checklist item	Location where item is reported
		including how many reviewers collected data from each report, whether they worked independently, any processes for obtaining or confirming data from study investigators, and if applicable, details of automation tools used in the process.	Study Quality Assessment and Data Extraction, Statistical Analysis.
Data items	10a	List and define all outcomes for which data were sought. Specify whether all results that were compatible with each outcome domain in each study were sought (e.g. for all measures, time points, analyses), and if not, the methods used to decide which results to collect.	Materials and Methods, Study Quality Assessment and Data Extraction.
	10b	List and define all other variables for which data were sought (e.g. participant and intervention characteristics, funding sources). Describe any assumptions made about any missing or unclear information.	Materials and Methods, Study Quality Assessment and Data Extraction.
Study risk of bias assessment	11	Specify the methods used to assess risk of bias in the included studies, including details of the tool(s) used, how many reviewers assessed each study and whether they worked independently, and if applicable, details of automation tools used in the process.	Materials and Methods, Study Quality Assessment and Data Extraction.
Effect measures	12	Specify for each outcome the effect measure(s) (e.g. risk ratio, mean difference) used in the synthesis or presentation of results.	Materials and Methods, Statistical Analysis.
Synthesis methods	13a	Describe the processes used to decide which studies were eligible for each synthesis (e.g. tabulating the study intervention characteristics and comparing against the planned groups for each synthesis (item #5)).	Results, Characteristics and Quality of Included Studies.
	13b	Describe any methods required to prepare the data for presentation or synthesis, such as handling of missing summary statistics, or data conversions.	Materials and Methods, Statistical Analysis.
	13c	Describe any methods used to tabulate or visually display results of individual studies and syntheses.	Materials and Methods, Statistical Analysis.
	13d	Describe any methods used to synthesize results and provide a rationale for the choice(s). If meta-analysis was performed, describe the model(s), method(s) to identify the presence and extent of statistical heterogeneity, and software package(s) used.	Materials and Methods, Statistical Analysis.
	13e	Describe any methods used to explore possible causes of heterogeneity among study results (e.g. subgroup analysis, meta-regression).	Materials and Methods, Statistical Analysis.
	13f	Describe any sensitivity analyses conducted to assess	Materials and Methods, Statistical Analysis.

Section and Topic	Item #	Checklist item	Location where item is reported
		robustness of the synthesized results.	
Reporting bias assessment	14	Describe any methods used to assess risk of bias due to missing results in a synthesis (arising from reporting biases).	Materials and Methods, Statistical Analysis.
Certainty assessment	15	Describe any methods used to assess certainty (or confidence) in the body of evidence for an outcome.	Materials and Methods, Statistical Analysis.
RESULTS			
Study selection	16a	Describe the results of the search and selection process, from the number of records identified in the search to the number of studies included in the review, ideally using a flow diagram.	Results, Study Selection, Figure 1.
	16b	Cite studies that might appear to meet the inclusion criteria, but which were excluded, and explain why they were excluded.	Results, Study Selection.
Study characteristics	17	Cite each included study and present its characteristics.	Results, Characteristics of Included Studies, Table 2.
Risk of bias in studies	18	Present assessments of risk of bias for each included study.	Results, Characteristics of Included Studies, Table 1.
Results of individual studies	19	For all outcomes, present, for each study: (a) summary statistics for each group (where appropriate) and (b) an effect estimate and its precision (e.g. confidence/credible interval), ideally using structured tables or plots.	Results, Primary Prognostic Association Between Serum Ferritin and Overall Survival, Figure 2.
Results of syntheses	20a	For each synthesis, briefly summarise the characteristics and risk of bias among contributing studies.	Results, Characteristics of Included Studies,Prognostic Association Between Serum Ferritin and Overall Survival.
	20b	Present results of all statistical syntheses conducted. If meta-analysis was done, present for each the summary estimate and its precision (e.g. confidence/credible interval) and measures of statistical heterogeneity. If comparing groups, describe the direction of the effect.	Results, Prognostic Association Between Serum Ferritin and Overall Survival.
	20c	Present results of all investigations of possible causes of heterogeneity among study results.	Results, Prespecified Subgroup Analyses.
	20d	Present results of all sensitivity analyses conducted to assess the robustness of the synthesized results.	Results, Sensitivity Analysis.
Reporting biases	21	Present assessments of risk of bias due to missing results (arising from reporting biases) for each synthesis assessed.	Results, Publication Bias.
Certainty of evidence	22	Present assessments of certainty (or confidence) in the body of evidence for each outcome assessed.	Results, Publication Bias, Figure 4.
DISCUSSION			

Section and Topic	Item #	Checklist item	Location where item is reported
Discussion	23a	Provide a general interpretation of the results in the context of other evidence.	Discussion.
	23b	Discuss any limitations of the evidence included in the review.	Strengths, Novel Contributions, and Limitations, Study Limitations.
	23c	Discuss any limitations of the review processes used.	Strengths, Novel Contributions, and Limitations, Study Limitations.
	23d	Discuss implications of the results for practice, policy, and future research.	Strengths, Novel Contributions, and Limitations, Study Limitations.
OTHER INFORMATION			
Registration and protocol	24a	Provide registration information for the review, including register name and registration number, or state that the review was not registered.	Materials and Methods, Study Design and Data Sources.
	24b	Indicate where the review protocol can be accessed, or state that a protocol was not prepared.	Abstract, Experimental Design.
	24c	Describe and explain any amendments to information provided at registration or in the protocol.	None
Support	25	Describe sources of financial or non-financial support for the review, and the role of the funders or sponsors in the review.	None
Competing interests	26	Declare any competing interests of review authors.	None
Availability of data, code and other materials	27	Report which of the following are publicly available and where they can be found: template data collection forms; data extracted from included studies; data used for all analyses; analytic code; any other materials used in the review.	Materials and Methods, Data Availability.

From: Page MJ, McKenzie JE, Bossuyt PM, Boutron I, Hoffmann TC, Mulrow CD, et al. The PRISMA 2020 statement: an updated guideline for reporting systematic reviews. BMJ 2021;372:n71. doi: 10.1136/bmj.n71. This work is licensed under CC BY 4.0. To view a copy of this license, visit https://creativecommons.org/licenses/by/4.0/

2. Full Database-Specific Search Strategies

PubMed

(((("Neoplasms"[Mesh]) OR ((((((((((((((Tumors[Title/Abstract]) OR (Neoplasia[Title/Abstract])) OR (Neoplasias[Title/Abstract])) OR (Neoplasm[Title/Abstract])) OR (Tumor[Title/Abstract])) OR (Cancer[Title/Abstract])) OR (Cancers[Title/Abstract])) OR (Malignant Neoplasm[Title/Abstract])) OR

(Malignancy[Title/Abstract])) OR (Malignancies[Title/Abstract])) OR (Malignant Neoplasms[Title/Abstract])) OR (Neoplasm, Malignant[Title/Abstract])) OR (Neoplasms, Malignant[Title/Abstract])) OR (Benign Neoplasms[Title/Abstract])) OR (Neoplasms, Benign[Title/Abstract])) OR (Neoplasm, Benign[Title/Abstract])) OR (Benign Neoplasm[Title/Abstract]))) AND (("Ferritins"[Mesh]) OR ((((Ferritin[Title/Abstract]) OR (Basic Isoferritin[Title/Abstract])) OR (Isoferritin, Basic[Title/Abstract])) OR (Isoferritin[Title/Abstract])))) AND (("Hyperferritinemia"[Mesh]) OR ((((((((Hyperferritinemias[Title/Abstract]) OR (Elevated Serum Ferritin[Title/Abstract])) OR (Serum Ferritin, Elevated[Title/Abstract])) OR (Raised Serum Ferritin[Title/Abstract])) OR (Serum Ferritin, Raised[Title/Abstract])) OR (Ultrahyperferritinemia[Title/Abstract])) OR (Dysmetabolic Hyperferritinemia[Title/Abstract])) OR (Hyperferritinemia, Dysmetabolic[Title/Abstract])))) AND (("Prognosis"[Mesh]) OR (((((Prognoses[Title/Abstract]) OR (Prognostic Factors[Title/Abstract])) OR (Prognostic Factor[Title/Abstract])) OR (Factor, Prognostic[Title/Abstract])) OR (Factors, Prognostic[Title/Abstract])))

Embase

（'ferritin'/exp OR ferritin OR 'serum ferritin':ab,ti）AND（'neoplasms'/exp OR neoplasms OR 'cancer':ab,ti OR 'malignancy':ab,ti）AND（'prognostic factors':ab,ti OR 'prognostic factor':ab,ti OR 'prognostic':ab,ti OR prognostic AND factors）

Cochrane Library

(Neoplasms/MeSH OR (Neoplasms):ab,ti,kw OR (Cancer):ab,ti,kw OR (Malignancy):ab,ti,kw) AND (Serum Ferritin/MeSH OR (Serum Ferritin):ab,ti,kw OR (Ferritin):ab,ti,kw) AND （ Prognoses/MeSH OR (Prognoses):ab,ti,kw OR (Prognostic Factors):ab,ti,kw OR (Prognostic Factor):ab,ti,kw OR (Factor):ab,ti,kw OR (Prognostic):ab,ti,kw）

CNKI

((SU=血清铁蛋白 OR SU=铁蛋白) AND (SU=癌 OR SU=恶性肿瘤 OR SU=肿瘤) AND (SU=预后 OR SU=生存期 OR SU=预后因素 NOT SU=动物实验 OR SU=

综述))

Wanfang Data

主题:(血清铁蛋白 铁蛋白) and 主题:(癌 恶性肿瘤 肿瘤) and 主题:(预后 生存期 预后因素)

3. Extracted Study Data and Review Manager Project File

All extracted data from the 10 included studies, as well as the complete Review Manager 5.4 project file (.rm5) and Stata 18.0 project file (.do) containing all statistical analyses, forest plots, and subgroup analyses, are available from the corresponding author upon reasonable request. The project file can be opened with Review Manager 5.4 (Cochrane Collaboration) and Stata 18.0 to fully reproduce all results presented in this manuscript.
