# Supplementary material for: Serum ferritin as a non-invasive prognostic biomarker in cancer: a systematic review and meta-analysis
Source: Front Oncol. 2026 Aug 4;16:1892172. doi: 10.3389/fonc.2026.1892172 (PMC13481198; doi:10.3389/fonc.2026.1892172)
Supplement: Supplementary file 2 [file Table1.pdf]

Table S1. Results of the Univariate REML Meta-Regression

covariate	Unitofcomparison	k	β（SE）	RHR（95%CI）	*P*	MetaR²
Cohort sample size	For every additional 100 cases	10	-0.021（0.012）	0.979 (0.957–1.002)	0.073	N.A.
Male proportion	For every additional 10 percentage points	9	-0.002（0.063）	0.998 (0.882–1.128)	0.969	N.A.
Year of Publication	For every additional 5 years	10	-0.033（0.079）	0.967 (0.829–1.129)	0.673	N.A.
SF cutoff value	For every increase of 100 ng/mL	8	-0.019（0.027）	0.981 (0.930–1.034)	0.477	N.A.
Blood collection and evaluation time point	Before treatment/evaluation vs after treatment/during treatment	10	-0.145（0.169）	0.865 (0.622–1.204)	0.390	N.A.
Tumor Type	Hematologic tumors vs solid tumors	10	0.144 (0.143)	1.155 (0.872-1.529)	0.315	N.A.

Note: The ratio of hazard ratios(RHR) equals exp(β). In the constant model, the heterogeneity variance (τ^2) is zero, implying that no between-study variance is attributable to covariates; thus, the meta-regression R-squared (Meta R^2) is reported as not applicable (N.A.). K indicates the number of included studies, β refers to the regression coefficient, and SE denotes the standard error.

Table S2. Clinical Subgroup Analyses and Interaction Tests

Dimension	Subgroup	k	Pooled HR (95% CI)	Within-group *P*	Interaction *P*
Sampling/assessment timing	Pre-index treatment/assessment	8	1.721 (1.466-2.021)	<0.001	0.390
Sampling/assessment timing	Post-treatment/in-treatment	2	1.989 (1.491–2.655)	<0.001	0.390
Tumor type	Hematologic malignancy	6	1.911 (1.570-2.325)	<0.001	0.315
Tumor type	Solid tumor	4	1.654 (1.353-2.023)	<0.001	0.315

Table S3. Robustness Analyses of Moderator Coding

Moderator	Complete-case or strict model	Alternative model	Conclusion
Male proportion	Complete case: k=9; β=-0.002; *P*=0.969	Median imputation + missing indicator: k=10; β=-0.002; *P*=0.969	Consistent direction and inference
SF cutoff	Strict model: k=8; β=-0.019; *P*=0.477	Sensitivity model: k=9; β=-0.011; *P*=0.660	No significant effect modification in either model
